# Absence of *Helicobacter pylori* high tetracycline resistant 16S rDNA AGA926-928TTC genotype in gastric biopsy specimens from dyspeptic patients of a city in the interior of São Paulo, Brazil

**DOI:** 10.1186/1471-230X-12-49

**Published:** 2012-05-17

**Authors:** Rodrigo Buzinaro Suzuki, Cristiane Maria Almeida, Márcia Aparecida Sperança

**Affiliations:** 1 Department of Genotyping, Hemocenter, Marilia Medical School, Marilia, SP, Brazil; 2 Department of Molecular Biology, Marilia Medical School, Marilia, SP, Brazil; 3 Center of Natural and Human Sciences, Universidade Federal do ABC, Rua Santa Adélia, 166 Bloco A, Torre 3, 6° andar, Sala 625, CEP 09210-170 Bairro Bangu, Santo André, SP, Brazil

**Keywords:** *Helicobacter pylori*, Tetracycline resistance, *Helicobacter pylori* 16 S rDNA, Nucleic acid based diagnosis, *Helicobacter pylori* 16 S rDNA polymorphism

## Abstract

**Background:**

Treatment effectiveness of *Helicobacter pylori* varies regionally and is decreasing worldwide, principally as a result of antibiotic resistant bacterium. Tetracycline is generally included in second line *H. pylori* eradication regimens. In Brazil, a high level of tetracycline resistance (TetR) is mainly associated with AGA926-928TTC 16 S rDNA nucleotide substitutions. As *H. pylori* culture is fastidious, we investigated the primary occurrence of *H. pylori* 16 S rDNA high level TetR genotype using a molecular approach directly on gastric biopsies of dyspeptic patients attending consecutively at Hospital das Clinicas of Marilia, São Paulo, Brazil.

**Methods:**

Gastric biopsy specimens of 68 peptic ulcer disease (PUD) and 327 chronic gastritis (CG) patients with a positive histological diagnosis of *H. pylori* were investigated for TetR 16 S rDNA genotype through a molecular assay based on amplification of a 16 S rDNA 545 bp fragment by polymerase chain reaction and H*infI* restriction fragment length polymorphism (PCR/RFLP). Through this assay, AGA926-928TTC 16 S rDNA TetR genotype resulted in a three DNA fragment restriction pattern (281, 227 and 37 bp) and its absence originated two DNA fragments (264 and 281 bp) due to a 16 S rDNA conserved H*inf I* restriction site.

**Results:**

The 545 bp 16 S rDNA PCR fragment was amplified from 90% of gastric biopsies from histological *H. pylori* positive patients. H*infI* RFLP revealed absence of the AGA926–928TTC *H. pylori* genotype and PCR products of two patients showed absence of the conserved 16 S rDNA H*infI* restriction site. BLASTN sequence analysis of four amplicons (two conserved and two with an unpredicted H*infI* restriction pattern) revealed a 99% homology to *H. pylori* 16 S rDNA from African, North and South American bacterial isolates. A nucleotide substitution abolished the conserved H*infI* restriction site in the two PCR fragments with unpredicted H*infI* RFLP, resulting in an E*coRI* restriction site.

**Conclusions:**

*H. pylori* AGA926-928TTC 16 S rDNA gene substitutions were not found in our population. More research is required to investigate if *H. pylori* TetR has a different genetic background in our region and if the nucleotide substitutions of the uncultured *H. pylori* 16 S rRNA partial sequences have biological significance.

## Background

It is widely accepted that *Helicobacter pylori*, a Gram negative microaerophilic bacterium, is associated with several digestive tract diseases such as chronic gastritis, peptic and duodenal ulcers, gastric cancer and lymphoproliferative disorders [1]. There is no standardized treatment regimen for *H.pylori* infection [2] and once the bacterium is detected in altered gastric mucosa, the indicated treatment consists of a triple antibiotic regimen including metronidazole, clarithromycin, amoxicillin, tinidazole, tetracycline and fluoroquinolones associated with a proton pump inhibitor such as omeprazole, lansoprazole or pantoprazole [3-5], according to antibiotic prescription policies for local medical care.

*H. pylori* eradication rates with a number of combined agents and regimens are close to 80% [6,7], varying from country to country and regionally within countries [8]. Several factors contribute to this low rate of *H. pylori* healing including the inefficiency of the antibiotic penetration in the gastric mucosa, inactivation of the antibiotic by the acid secretion of the stomach [9], lack of patient compliance [10] and principally, emergency cases and increase in *H. pylori* antibiotic resistant strains [11]. Thus, regional *H. pylori* resistance surveillance is of great importance for test and treatment strategies.

In Brazil, a country of continental dimensions, the majority of practicing clinicians include tetracycline in a second line treatment regimen after failure of the classical triple regimen composed of claritromycin, amoxicillin and a proton pump inhibitor for seven days to overcome *H. pylori* infection [12]**.**

*H. pylori* resistance to tetracycline (TetR) is low in most countries [13-15], conversely, in Latin America, according to a small number of studies, it has been shown to be high in Chile [16] and in Brazil [17]. Moreover, for some years the incidence of TetR has been increasing [15,18-21]. Accordingly, considering the clinical importance of primary *H. pylori* resistance to antibiotics, it should be considered regionally before being included in eradication regimens.

The gold standard method for determination of *H. pylori in vitro* susceptibility to antibiotics corresponds to the isolation of the microorganism by culture. However, because of the slow growth and the particular requirements of *H. pylori* culture, this approach is not reliable for use in most routine clinical laboratories, principally in developing countries. Hence, molecular tests targeting resistance associated gene mutations directly from biopsy specimens have the potential for use in large scale studies [22-25].

The molecular mechanism of TetR consists of its binding to a specific 16 S rRNA region, interacting stoically with the aminoacyl-tRNA transferase to the A site of the ribosome. This binding site has been defined by atomic resolution in ribosomes of *Thermus thermophilus* being formed by two domains of the 16 S rRNA fraction consisting of helix 34 and the loop next to helix 31 [26,27]. In *H. pylori* isolates, the high degree of TetR is mainly due to three base mutations, from AGA926-928 to TTC, in the 16 S rRNA genes rrnA/B [28,29]. Mutations in one or two of these positions result in a low level of TetR [30,31].

In Brazil, studies performed on patients from Bragança Paulista, São Paulo, showed that triple AGA926-928 to TTC mutations are found in all TetR *H. pylori* isolates [32]. Thus, using a molecular approach based on a polymerase chain reaction associated with restriction fragment length polymorphism (PCR-RFLP) assay, we investigated the primary incidence of *H. pylori* high level TetR directly in gastric biopsy specimens obtained from dyspeptic patients submitted to gastroscopy at Hospital das Clinicas of Marilia, São Paulo, Brazil, from January 2003 to July 2006.

## Results and discussion

Gastric disease outcome of 1102 patients attending the gastroenterology outpatient clinic of Hospital das Clínicas of Marília was investigated by endoscopy and histopathology. Endoscopic finding of peptic or duodenal ulcer disease (PUD) were present in 119 patients. Different degrees of chronic gastritis (CG) were observed by histopathology in 693 patients and other alterations corresponding mostly to gastroesophageal reflux disease (GERD) and normal gastric mucosa, were found in 290 patients. Some patients presented more than one alteration, with the most severe pathology being considered in the analysis.

Detection of *H. pylori* was performed directly from biopsy specimens by histology, the gold standard *H. pylori* diagnostic test employed in our clinical routine which together with histopathological analysis is used to decide for *H. pylori* eradication therapy. Of 119 PUD, 693 CG and 290 GERD samples, 76, 359 and 2, respectively, were positive for *H. pylori* by histology.

Once detected in gastric mucosa, a classical *H. pylori* eradication triple regimen is prescribed in our gastroenterology health care clinics. When first choice regimen therapy fails to eradicate *H. pylori*, a second line regimen containing tetracycline is the most indicated. *H. pylori* antibiotic TetR varies regionally, being very low in Europe and North America [2,33,34]. However, in Asia [15,19,35] and Latin America, including Brazil [16,32], a high rate of *H. pylori* TetR has been found. Thus, in order to improve the choice of *H. pylori* associated disease therapy, principally in case of first eradication failure, we investigated the regional high level of *H. pylori* TetR using a molecular approach based on PCR and RFLP directly from the same gastric biopsy used for rapid urease test. Only biopsy samples from the patients with positive *H. pylori* diagnosis by histology were included. A 545 bp *H. pylori* 16SrDNA PCR fragment was obtained from 89.5% (68/76) and 91.1% (327/359) of gastric biopsies from PUD and CG patients, respectively. As both tests were performed on a single and different gastric biopsy and *H. pylori* infection presents a focal characteristic of infection [36], to improve sensitivity of this method, multiple biopsy sampling is recommended.

Sequentially, in order to detect the major related point mutations, AGA to TTC at the positions 926, 927 and 928 of the *H. pylori* 16 S rDNA associated with a high level of TetR, and a unique genotype characterized in Brazilian TetR *H. pylori* isolates [32], the 545 bp 16 S rDNA PCR fragment was restricted with H*infI*. In this *H. pylori* 16 S rDNA amplicon there is a conserved H*infI* restriction site, which provides an internal control of enzyme digestion, resulting in a two DNA fragment restriction pattern, when the triple AGA926-928TTC nucleotide substitution is absent. Of 395 PCR samples, 393 presented the two DNA fragment restriction pattern and two PCR products obtained from a PUD (Hp16S563Mar) patient and a CG (Hp16S587bp) patient were not digested by H*infI*. The high tetracycline resistant AGA926-928TTC genotype dependent on *H. pylori* 16 S rDNA was not present in our population. These results can be indicative of *H. pylori* high level TetR absence or that in our region other 16 S rDNA nucleotide substitutions or different genetic factors are involved in tetracycline resistance, as found by other studies [21]. More research has to be carried out to confirm or exclude these hypotheses.

In order to confirm the specificity of the 545 bp *H. pylori* 16 S rDNA PCR products amplified from gastric biopsies, the 545 bp PCR fragments obtained from two *H. pylori* positive PUD patients used as controls in PCR reactions (Hp16S248Mar and Hp16S644Mar), and the 545 bp PCR fragments with unpredicted H*infI* restriction pattern named Hp16S563Mar and Hp16S587bp, were sequenced and analyzed by basic BLASTN search [37]. All four sequences presented 99% homology to the 16 S rDNA from West and South African, South and North American *H.pylori* isolates. The point mutations found in each analyzed PCR sequence compared to the *H. pylori* 16 S rDNA reference sequences presenting higher homology to the obtained amplicons are summarized in Table 1. Abolishment of the H*infI* restriction site of Hp16S563 and 587Mar resulted from a nucleotide substitution at position 958, originating in an E*coRI* restriction site. Thus, the nucleotide substitutions in these 16 S 545 bp PCR fragments were confirmed by E*coRI* restriction analysis (data not shown). The biological significance of nucleotide substitutions found in our 16 S uncultured *H. pylori* PCR fragments needs to be investigated. 

**Table 1:** **Comparison of* Helicobacter pylori *545 bp 16 S rDNA nucleotide polymorphisms among references and uncultured bacterium strains**

***H. pylori*strain+**	**16 S rDNA nucleotide positions#**							
	**926-928**	**947**	**954-959**	**981**	**988**	**1092**	**1093**	**1097**
**SouthAfrica07**	AGA	A	GAATCC	A	T	T	C	G
**2017WestAfrica**	AGA	A	GAATCC	A	T	T	C	G
**EU544200USA**	AGA	A	GAATCC	A	T	T	T	G
**Puno/Peru**	AGA	A	GAATCC	G	C	T	C	G
**Hp16S248Mar**	AGA	G	GAATCC	A	T	T	C	A
**Hp16S644Mar**	AGA	A	GAATCC	A	T	G	C	G
**Hp16S563Mar**	AGA	A	GAATTC	A	T	T	T	G
**Hp16S587Mar**	AGA	A	GAATTC	G	C	T	C	G

# based on *H. pylori* reference strain 26995; + accession numbers of the *H. pylori* sequence strains for SouthAfrica07, 2017WestAfrica, EU544200USA, Puno/Peru, Hp16S248Mar, Hp16S644mar, Hp16S563Mar and Hp16S567Mar are, respectively: [Genbank:CP002336.1, Genbank:CP002571.1, Genbank:EU544200.1, Genbank:CP002982.1, Genbank:JQ315410, Genbank:JQ315411, Genbank:JQ315412 and Genbank:JQ315413].

## Conclusions

The high level TetR *H. pylori* genotype dependent on AGA926-929TTC 16 S rDNA gene substitutions was not found in our population. More research is required to investigate if *H. pylori* high rate TetR is absent or if it is associated with a different bacterial genetic background in our region. Also, the biological significance of the unpredicted nucleotide substitutions of the Marilia uncultured *H. pylori* 16 S rDNA partial sequence needs further investigation.

## Methods

### Patients

1102 adult patients resident in Marilia city, São Paulo State, Brazil, aged 19 to 91 years, who had consecutively undergone esophagogastroduodenoscopy (EGD) for upper abdominal pain or dyspeptic symptoms from January 2003 through July 2006 at the gastroenterology outpatient clinic of the Hospital das Clínicas of Marília Medical School, were enrolled in this study.

### Endoscopy, biopsies

The EGD was accomplished by fibroendoscope (GIF-XP20, GIF-XQ20) or video-endoscope (GIF-100) both from Olympus. Gastric or duodenal ulcer diagnosis was defined by endoscopy and two fragments of the antrum were collected to perform the rapid urease and histopathological tests. The biopsy used for the rapid urease test was further submitted to DNA extraction. The protocol used is in agreement with the Helsinki Declaration and was approved by the Ethical Committee in Human Research from Marilia Medical School, under reference number 388/01.

### Histology

One antral specimen was fixed in formol solution at 10% and embedded in paraffin. Sections were Giemsa stained for *H. pylori* evaluation and were stained with hematoxilin and eosin for assessment of histopathologic alterations [38].

### DNA extraction and Polymerase chain reaction

Polymerase chain reaction and restriction analysis were set up with the same biopsy used for the rapid urease test. This was submitted to DNA extraction with the employment of the GFx DNA extraction kit purchased from Amersham/Pharmacia Biotech, following the manufacturer’s instructions. DNA was quantified in agarose gel electrophoresis using the Invitrogem low mass ladder and 50-100ηg were used in the PCR reactions with the primer set Hp16Sr1 (sense),): 5′ AAC ATT ACT GAC GCT GAT TG 3′; Hp16S r2 (antisense): 5′ TGG CTC CAC TTC GCA GTA TT 3′, which amplify a conserved fragment of 545 bp corresponding to the *H. pylori* 16 S rRNA gene between nucleotide positions 700 and 1245 (numbered according to the rrnA gene of *H. pylori* strain 26695), modified from [32]. In all PCR reactions a negative and a positive control were used corresponding to, respectively, sterile water and two different urease *H. pylori* positive gastric biopsies from PUD patients. PCR condition was 94°C 5′ followed by 40 cycles of 94°C 1′/55°C 1′/72°C 1′ and one cycle at 72°C 7′, with a total volume of 25 μl containing 1x PCR buffer, 200 μM dNTPs, 2.0 mM MgCl_2_, 1μM oligoHp16Sr1, 1μM oligo Hp16Sr2, 1.25 U Taq DNA Polymerase Platinum Brazil (Invitrogen), 2.5% DMSO, 50ηg DNA. The PCR products were resolved in 1.5% agarose gels stained with ethidium bromide and photographed under UV light.

### Restriction and sequencing analysis

The 16 S rDNA 545 bp amplicons obtained by PCR from biopsy specimens were digested with H*inf* I (biolab – New England) according to the manufacturer’s instructions. The predictable restriction pattern for tetracycline susceptible *H. pylori* strains corresponds to two fragments of 264 and 281 bp and for *H. pylori* strains with high tetracycline resistance corresponds to three fragments of 281, 227 and 37 bp. The products of restriction analysis were resolved in 8% acrylamide gels, stained with ethidium bromide and photographed under UV light. 16 S rRNA 545 bp PCR amplicons from the two *H. pylori* positive control gastric biopsies (Hp16S248Mar and 644Mar) and the two amplicons presenting unexpected H*inf* I restriction patterns (Hp16S563Mar and Hp16S587bp) were submitted to sequencing with DyeTM Terminator v3.0 cycle Sequencing Ready Reaction kit and an ABI-3100 machine purchased from Applied Biosystem, according to the manufacturer’s instructions. Nucleotide sequence determination was performed in duplicate and comparative analysis was carried out by basic nucleotide BLAST alignment [37].

## Abbreviations

PUD: Peptic ulcer disease; CG: Chronic gastritis; GERD: Gastroesophageal reflux disease; PCR: Polymerase chain reaction; RFLP: Restriction fragment length polymorphism; TetR: Tetracycline resistance.

## Competing interests

We do not have any to declare.

## Author’s contributions

RBS and CMA carried out the molecular studies and contributed to the acquisition and interpretation of data; MAS designed the experiments, contributed to data analysis and drafted the manuscript. All authors read and approved the final manuscript.

## Pre-publication history

The pre-publication history for this paper can be accessed here:

http://www.biomedcentral.com/1471-230X/12/49/prepub
